# The successive emergence of ERVL-MaLRs in primates

**DOI:** 10.1093/ve/vead072

**Published:** 2023-12-04

**Authors:** Zheng Zuo

**Affiliations:** School of Life Science and Technology, Southeast University, Nanjing 210096, China

**Keywords:** ERVL-MaLR, MST, THE1B, THE1A, ZNF430, ZNF100, evolutionary arms race

## Abstract

Although the ERVL-mammalian-apparent LTR retrotransposons (MaLRs) are the fourth largest family of transposable elements in the human genome, their evolutionary history and relationship have not been thoroughly studied. In this study, through RepeatMasker annotations of some representative species and construction of phylogenetic tree by sequence similarity, all primate-specific MaLR members are found to descend from MLT1A1 retrotransposon. Comparative genomic analysis, transposition-in-transposition inference, and sequence feature comparisons consistently show that each MaLR member evolved from its predecessor successively and had a limited activity period during primate evolution. Accordingly, a novel MaLR member was discovered as successor of MSTB1 in Tarsiiformes. At last, the identification of candidate precursor and intermediate THE1A elements provides further evidence for the previously proposed arms race model between ZNF430/ZNF100 and THE1B/THE1A. Taken together, this study sheds light on the evolutionary history of MaLRs and can serve as a foundation for future research on their interactions with zinc finger genes, gene regulation, and human health implications.

## Introduction

**Figure 1.**
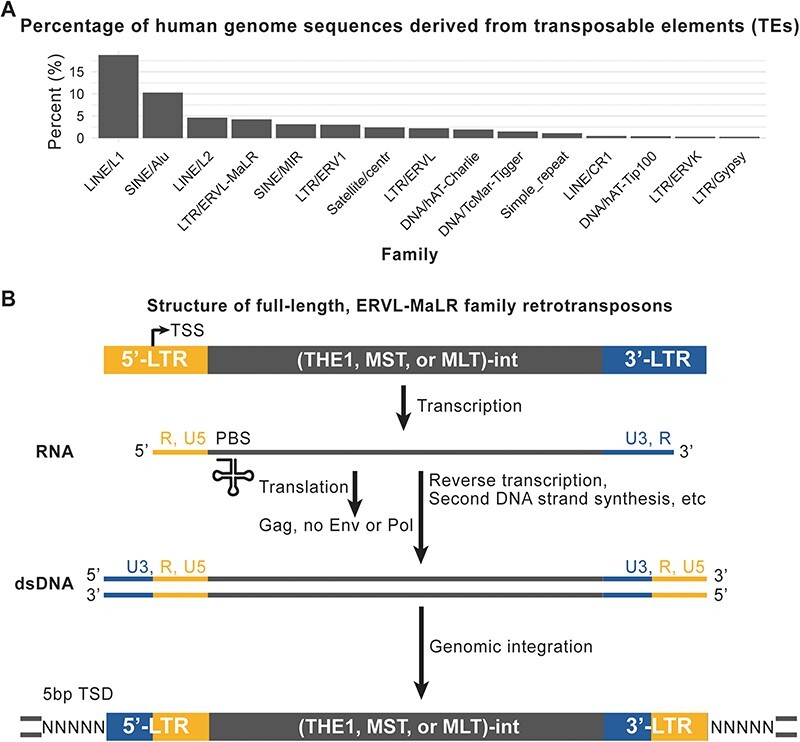
Background about ERVL-MaLR retrotransposons. A) Percentage of human genome that is derived from transposons; Only top 15 families are shown.B) The composition of ERVL-MaLR and the replication mechanism of LTR-retrotransposons. TSS: Transcription start site; PBS: Primer binding site; TSD: Target site duplication.

At least 40 per cent of the human genome sequences are estimated to derive from transposable elements throughout history, including LINE-1, SINE, ERV, and others (Hedges and Batzer 2005). One noteworthy family among these elements is ERVL-mammalian-apparent LTR retrotransposon (MaLR) (Paulson et al. 1985; Smit 1993), which currently makes up approximately 4 per cent of our genome (Fig. 1A). This family has been reported to be involved in various biological processes, such as placental gene expression (Cohen et al. 2011), formation of recombination hotspots (Bhérer, Campbell, and Auton 2017), X chromosome inactivation (Sun and Chadwick 2018), and cancer progression (Edginton-White et al. 2019; Chen et al. 2022). However, the evolutionary history of ERVL-MaLR, including the succession order and activity period of each member, has not been previously reported.As a sub-family of the ERV3 retrovirus, MaLR possesses two distinctive features. First, its internal regions only consist of one open reading frame for the putative gag gene, instead of env or pol (Fig. 1B). This implies that MaLR is unable to replicate and propagate independently. Second, the 5′and 3′ long terminal repeat (LTR) regions of MaLR contain more sequence variations compared to the internal sequences. Consequently, the current RepBase (Bao, Kojima, and Kohany 2015) and Dfam databases (Storer et al. 2021) classify the MaLR family into at least forty members based on the sequence content in the LTR regions, while the internal sequences are broadly classified into three classes: MLT-int, MST-int, and THE1-int. There might exist molecular mechanisms, such as the competition between retrotransposons and anti-viral factors in the host (Lukic and Chen 2011; Jacobs et al. 2014; Yang, Wang, and Macfarlan 2017), that have driven the rapid and continuous evolution of MaLR in its LTR regions.

There are several lingering questions regarding the evolution of MaLR. Firstly, it remains unclear when each member evolved from its predecessor and whether or not each member had a limited activity period in history, particularly during the primate lineage. Secondly, the driving forces behind the sequence changes between each member are still unknown. There are two possible scenarios to consider. In the first scenario, all members evolved concurrently and independently from each other, meaning that the observed sequence differences between different members are merely random or coincidental. On the other hand, the second scenario proposes that each member evolved from a specific predecessor and was actively propagating for a limited period. Under this circumstance, the sequence changes in each MaLR from its predecessor are likely crucial adaptations to the host environment during that particular historical period. Without evidence to distinguish between these two scenarios, further research on the evolutionary driving forces cannot proceed.For instance, two MaLR members, THE1B and THE1A, were previously found to be targeted by two simian-specific, paralogous KRAB-zinc finger (ZNF) genes, ZNF430 and ZNF100, respectively (Imbeault, Helleboid, and Trono 2017). Compared to the ZNF430 binding site within THE1B element, the corresponding locus in THE1A is specifically bound by ZNF100 and contains only 1bp deletion, termed as the 204del event (defined on the consensus sequence of THE1B). It is highly likely that this 204del event facilitated THE1A’s evasion from ZNF430’s silencing and contributed to THE1A’s rapid expansion, ultimately leading to its succession over THE1B. However, ZNF100 later silenced THE1A, creating a continuing evolutionary arms race or battle between THE1B/THE1A and ZNF430/ZNF100 (Zuo 2023). Nonetheless, this model assumes implicitly that THE1A directly evolved from THE1B, instead of any other MaLR member, which has not been demonstrated before. Moreover, if this model is accurate, there may exist some intermediate elements between THE1B and THE1A within the human genome today, which could explain the chronological order of sequence changes that occurred between the two MaLRs.

To address these questions, I employed a range of techniques, namely phylogenetic tree construction, comparative genomic analysis, transposition-in-transposition (TinT) inference, and sequence feature comparison to investigate the evolutionary history of all primate-specific MaLRs. Notably, the results of this study reveal that THE1A directly evolved from THE1B. It is worth mentioning that the sequence composition found in the intermediate elements between THE1B and THE1A indicates that the 204del event occurred immediately prior to the expansion of THE1A. This finding provides additional evidence supporting the notion that the battle between MaLRs and ZNFs is one fundamental force driving the continuous evolution of MaLRs.

## Results

### All primate-specific MaLRs descended from one lineage

**Figure 2.**
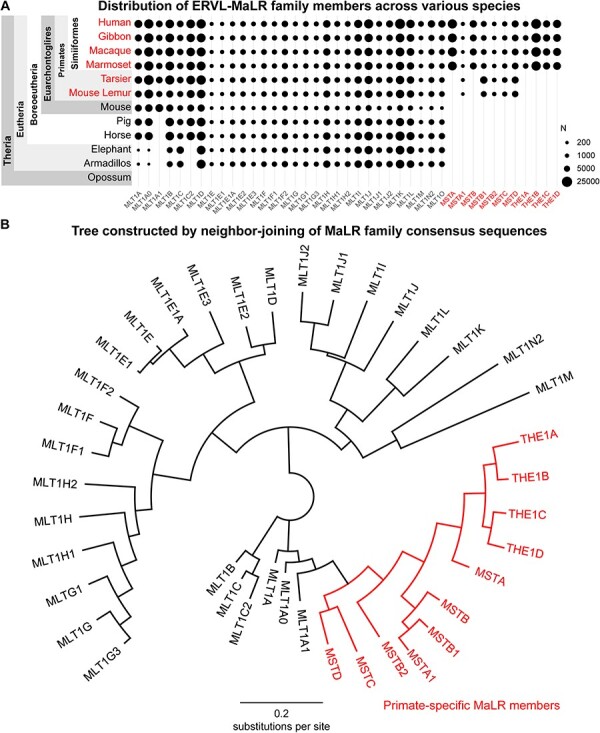
A) Distribution of MaLR members in some representative species according to RepeatMasker annotations provided by UCSC genome browser; Primates and primate-specific MaLRs are labelled red. B) Phylogenetic tree constructed by neighbor-joining of MaLR consensus sequences in LTR regions; parameters are described in Methods section.

MaLR (Smit 1993) is known to exclusively exist in mammals. To determine the distribution of MaLRs in different species, we can select representative species from each clade and compile public data from the RepeatMasker database (Fig. 2A). Note that only those MaLRs that exist in the human genome are listed, so it is likely that other non-human MaLRs in rodents or other species are not shown here.

Based on the current RepeatMasker annotation, eleven MaLR members, including all MST and THE1 sub-family members, are exclusively found in primates. All other members, except MLT1A1 and MLT1C2, have a widespread presence in eutherian genomes but are absent in opossum genome, indicating their ancestral origin in the common ancestor of placental mammals. MLT1A1 is exclusively found in Euarchontoglires, suggesting that it is originated in the common ancestor of primates and rodents and is younger than MLT1A and MLT1A0.Constructing a phylogenetic tree through neighbor-joining of the LTR consensus sequences of all MaLR members is achievable (Fig. 2B and Fig. S1). Remarkably, MaLR members can be grouped into three major branches, with all primate-specific MaLRs falling within a single branch, indicating their descent from a common lineage. The closest non-primate-specific neighbor to primate-specific MaLRs is MLT1A1, suggesting that all primate-specific MaLRs evolved from ancestral MLT1A1.

### MSTB, MSTA, and THE1-family retrotransposons emerged after the Simiiformes-Tarsiiformes split

Among the eleven primate-specific members, six of them (MSTB, MSTA, THE1D, THE1C, THE1B, and THE1A) are solely present in simian genomes and cannot be found in tarsier, mouse lemur, or bushbaby. This means that these six members emerged after the divergence of Simiiformes and Tarsiiformes, within the last 70 million years. Interestingly, five of these six members form a sub-branch, with MSTB being their closest neighbor in the adjacent sub-branch (Fig. 2B). It is probable that MSTB is the oldest member among these six MaLRs and evolved directly from another non-simian-specific MaLR, such as MSTB1 or MSTA1.

### MSTD, MSTC, MSTB2, and MSTA1 were primarily active before the Haplorhini-Strepsirrhini split

Since the RepeatMasker annotations were downloaded from UC Santa Cruz (UCSC) genome browser, the analysis could be confounded if RepeatMasker was run using different parameters or library versions for each genome. We need some orthogonal methods such as comparative genomics to verify the above results. The genomes of the mouse lemur, bushbaby, and tarsier are valuable resources as they provide insights into events that occurred during the early stages of primate evolution (Schmitz et al. 2016). It is known that the split between Haplorhini and Strepsirrhini happened before the split between Simiiformes and Tarsiiformes (Ziętkiewicz, Richer, and Labuda 1999; Hartig et al. 2013). In other words, the mouse lemur and bushbaby diverged from our common ancestor before the tarsier did.

**Figure 3.**
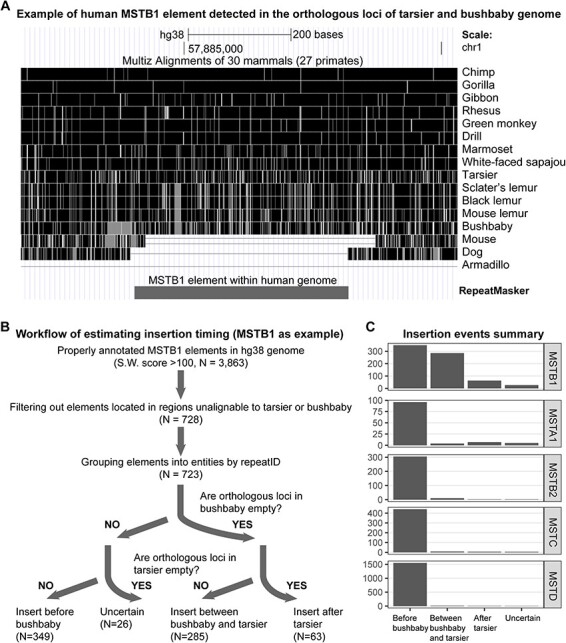
A) Example of one solo MSTB1 element in human genome (rectangle) which can be detected in the orthologous loci of tarsier and bushbaby genomes, indicating the corresponding insertion event happened before the Haplorhini-Strepsirrhini split. Vertical white lines indicate mismatch between human and corresponding specie. B) Workflow of estimating insertion timing. C) Insertion events summary when the same workflow was applied to each MaLR member.

For example, if a transposon insertion event occurred in the common ancestor of primates, we would expect the inserted element to be present in the human genome, as well as in the orthologous loci of the bushbaby and tarsier, simultaneously. This can be seen with the MSTB1 element shown in Fig. 3A. Similarly, any insertion event that occurred between the two aforementioned splits would only be detected in the human genome and the orthologous locus of the tarsier but not in the bushbaby or mouse lemur. This approach of identifying lineage-specific insertions through comparative genomics has been used in various studies before (Hardison and Miller 1993; Churakov et al. 2020; Kelly, Chitko-mckown, and Chuong 2022; Papon et al. 2023), and it relies on accurate repeat annotations and proper sequence alignment between the genomes of the studied species.

We can use this approach to estimate the relative timing of transpositions of five MaLRs: MSTD, MSTC, MSTB2, MSTA1, and MSTB1. The detailed workflow is illustrated in Fig. 3B, and the list of insertion events is included in the Supplemental Information. Since the bushbaby and tarsier diverged from the simian lineage more than 55 million years ago, only a small fraction of MaLR elements within the human genome and their flanking regions can be properly aligned to the orthologous loci of the tarsier and bushbaby (mouse lemur is not used here due to even lower alignment ratios). However, as long as the same consistent criteria are applied to analyze each of these five MaLR members (see Methods), it is still possible to draw reliable conclusions about their relative timing of insertions.

For MSTD, MSTC, MSTB2, and MSTA1, the majority of their insertions with proper alignment are detected in both bushbaby and tarsier simultaneously (Fig. 3C), indicating that they were primarily active in the common ancestor of primates and ceased replication before the Haplorhini-Strepsirrhini split. MSTB1 is unique because nearly half of its properly aligned insertions (285 out of 697) are only detectable in the tarsier and absent in the bushbaby (Fig. 3C). Overall, during the early stage of Haplorhini evolution, MSTB1 was the only active MaLR retrovirus.

### TinT inference reveals the approximate activity periods of MaLRs

**Figure 4.**
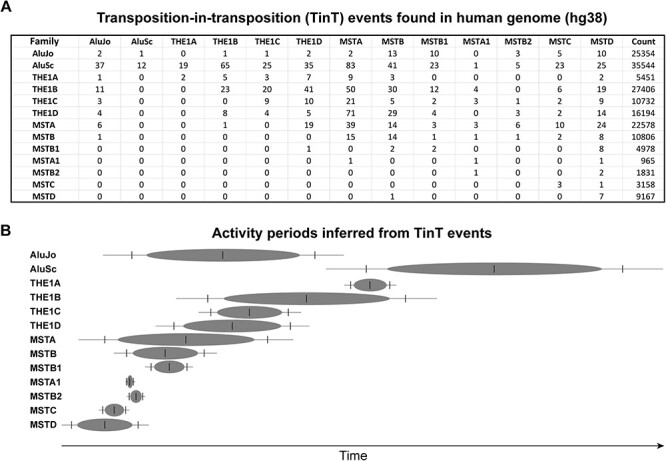
A) TinT events summary including AluJo, AluSc, and all primate-specific MaLRs in human genome. B) The oval center indicates the maximum of each activity period; the ends of each oval, the vertical lines and the ends of each line encompass 75%, 95%, and 99% of the inferred activity period along pseudo time respectively.

TinT inference is a clever method used to estimate the relative activity periods of different transposons in history (Churakov et al. 2010). This method is based on the concept of TinT, where if element A is nested within element B, the transposition of A should occur after the transposition of B. Given a collection of detected TinT events (Fig. 4A), it is possible to perform maximum likelihood estimation to infer the relative activity periods of different transposons along pseudo-time. One significant advantage of this approach is that it does not rely on the phylogenetic tree or molecular clock assumption. However, it does have practical limitations. In some cases, for retrotransposons like MSTB2 and MSTA1 that have a limited number of copies within the human genome (1,831 and 965, respectively), the inferred results may have a high degree of uncertainty due to the lack of TinT events with other transposons. A similar approach was developed by Giordano *et al*. (Giordano et al. 2007).

The original TinT inference paper (Churakov et al. 2010) demonstrated that two abundant groups of transposons, AluJo and AluSc, have distinct, non-overlapping activity periods. Specifically, AluJo was active around 75 million years ago, while AluSc was active around 45 million years ago. These transposons serve as excellent molecular markers and are included in current TinT analyses along with primate-specific MaLRs including their LTR and internal regions to infer their relative activity periods. First and foremost, each MaLR member had a limited activity period (Fig. 4B), which aligns with observations from comparative genomic analysis. Additionally, THE1 family members emerged after all other MST members, consistent with their simian-specific nature. Lastly, it is worth mentioning that THE1A is the youngest member among all MaLRs and only overlaps with THE1B. This reflects the fact that only THE1A-in-THE1B, THE1A-in-THE1C, and THE1A-in-THE1D events are detected but not vice versa (Fig. 4A).

### MSTB1 was succeeded by alternative MaLR member in Tarsiiformes

**Figure 5.**
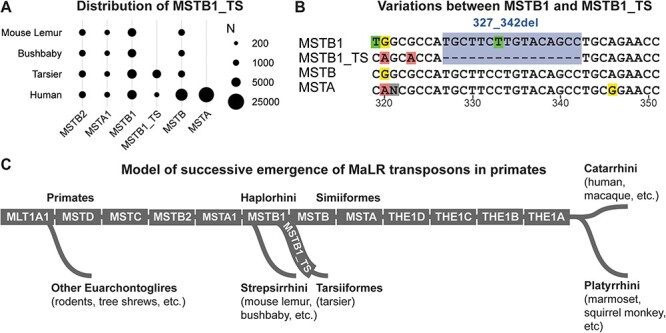
A) Distribution of MSTB1_TS and its close relatives in relevant species through RepeatMasker identification. B) Comparison of MSTB1_TS with MSTB1, MSTB, and MSTA; blue box represents the 16bp deletion event, which is uniquely detected in tarsier genome. C) Simplified model of successive emergence of MaLRs; The exact time scales are not shown.

The current results show that only MSTB1 was active during the early stage of Haplorhini evolution and MSTB emerged after the Simiiformes-Tarsiiformes split; thus, it is very likely alternative successor of MSTB1 exists in bushbaby or tarsier genome. To confirm this hypothesis, I conducted *de novo* repeat modeling and identification using the well-established RepeatModeler and RepeatMasker protocols, respectively (Flynn et al. 2020). Indeed, a new group of MSTB1-related repeats, named as MSTB1_TS, were predominantly found in tarsier genome (38,924 copies) but were an order of magnitude fewer in mouse lemur (127 copies), bushbaby (193 copies), or human (382 copies) as shown in Fig. 5A. Compared to MSTB1 and MSTB, this newly identified MaLR member contains a unique 16bp deletion (227_242del, Fig. 5B and Fig. S2). The close inspection of all reported MSTB1_TS elements reveals that this deletion feature exists almost exclusively in tarsier genome but not in mouse lemur, bushbaby, or human genome; thus, it remains to be determined whether this deletion event contributed to MSTB1_TS expansion in Tarsiiformes.

### Each primate-specific MaLR evolved from its direct predecessor successively

Both comparative genomic analysis and TinT inference demonstrate that each MaLR was only active for a limited period. It appears that, at any stage of primate evolution until approximately 45 million years ago, only one major MaLR was replicating, similar to the expansion of MSTB1 during the early stage of Haplorhini evolution. Based on this observation, we can propose a simplified model of MaLR evolution in the primate lineage, known as the model of successive emergence (Fig. 5C). According to this model, each MaLR member evolved from its direct predecessor and actively expanded for a certain period before being succeeded by another member or going extinct (THE1A).To validate the proposed succession order of this model (Fig. 5C), consensus sequence alignment was conducted for each MaLR and its close relatives, which included the direct predecessor, predecessor of predecessor, and successor (Fig. S3). If the proposed model is correct, there should be uniquely shared features between each member and its direct predecessor. Indeed, for each pair of MaLR and its predecessor, such shared features were identified. For example, a 35bp long feature (Positions 35 to 70, Panel 4 in Fig. S3) uniquely exists in MSTB2 and MSTA1, and a 37bp long feature (Positions 256 to 293, Panel 5 in Fig. S3) uniquely exists in MSTA1 and MSTB1. These findings confirm that MSTA1 is an intermediate member between MSTB2 and MSTB1 and is not directly related to MSTA. Therefore, it may be appropriate to consider renaming MSTA1 as MSTB1.5 or a similarly relevant name in future updates of the Dfam database.

### THE1A evaded the silencing of ZNF430 through 204del event right before its expansion

**Figure 6.**
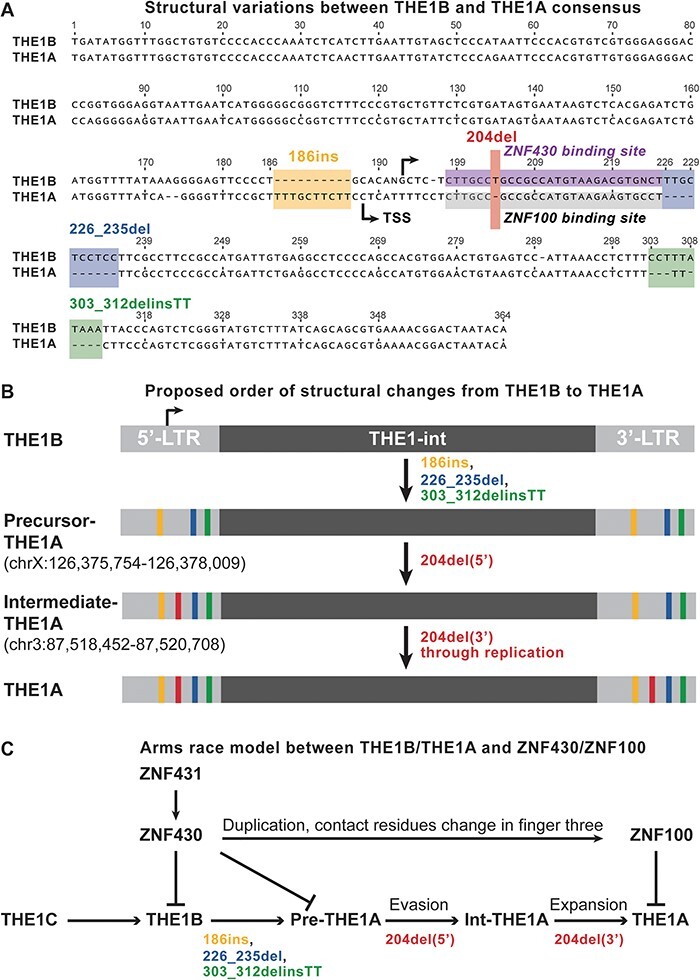
A) Structural variations between THE1B and THE1A consensus; ZNF430 and ZNF100 binding sites are labeled with different colors; Coordinates are defined based on THE1B consensus. B) Proposed order of structural changes from THE1B to THE1A; 204del(5’) and 204del(3’) indicate 204del on 5’ and 3’ LTR respectively. C) Arms race model between THE1B/THE1A and ZNF430/ZNF100; Pre-THE1A is short for Precursor-THE1A;Int-THE1A is short for Intermediate-THE1A.

The proposed succession order in Fig. 5C strongly supports the arms race model between THE1B/THE1A and ZNF430/ZNF100 mentioned in the Introduction section. However, when the THE1B and THE1A consensus sequences were aligned, it was observed that there were at least fifteen nucleotide substitutions and four structural variations (186ins, 204del, 226_235del, and 303_312delinsTT) between these two MaLRs (Fig. 6A). The 204del event is just one of these variations. It is unclear whether the evasion from ZNF430 through the 204del event truly drove the expansion of THE1A after THE1B.In the hypothesis, if a single mutation event is the main driving force behind the expansion of a new viral strain, we expect it to occur as the last mutation right before the viral expansion. As a result, only the mutations preceding this driver mutation would be commonly present in all progenies of the new strain. Although single-nucleotide substitutions might not be suitable for testing this hypothesis in the case of THE1 family retrotransposons, as their ‘molecular fossil’ in our genome has undergone more than 40 million years of changes, characteristic structural variations such as 204del could still be identified in most elements with confidence, provided that proper sequence alignment with orthologous species is performed.To determine the sequence of structural changes prior to THE1A expansion, a thorough examination of all human THE1A elements containing internal regions was carried out to identify intermediate elements that were expected to lack at least one of the aforementioned structural changes. Only two elements were found to fit this criterion: the Precursor-THE1A element and the Intermediate-THE1A (Fig. 6B). The Precursor-THE1A or Pre-THE1A element contains three indel events (186ins, 226_235del, and 303_312delinsTT) but not 204del in both its 5′ and 3′ LTRs. Interestingly, the Intermediate-THE1A or Int-THE1A contains not only the three indels in both LTRs but also 204del in its 5′ LTR. It is expected that THE1 follows the general replication mechanism of LTR retrotransposons (Finnegan 2012), where the R and U5 regions of the 5′ LTR are copied to the 3′ LTR and the U3 region of the 3′ LTR is copied to the 5′ LTR. Since 204del is located downstream of the transcription start site of the THE1A, as inferred through transcripts of IL23R, THOC5, and SQOR (Fig. 6A), 204del in 5′ LTR is expected to be copied to the 3′ LTR in the next round of replication. In other words, the progeny of Int-THE1A should contain all structural features observed in the THE1A consensus sequence in both LTRs. Sequence alignment of these two elements with their orthologous elements in other species, including apes (chimp, gorilla, and orangutan), old world monkeys (macaque), and new world monkeys (squirrel monkey), reveals that the observed structural features are highly conserved in simians (Figs. S4 and S5). It is quite likely that the Int-THE1A element is the founder element for THE1A, and 204del represents the final structural change just before THE1A’s expansion, fitting well with our hypothesis and the arms race model (Fig. 6C).

## Discussion

Existing ChIP-seq data show that besides ZNF430 and ZNF100, some other ZNFs, such as ZNF267 and ZNF776, target THE1C and MSTA, respectively (Imbeault, Helleboid, and Trono 2017). However, the full-length specificity profiles and recognition models of these ZNFs are unknown, so we currently have no knowledge on how they recognize and silence relevant MaLRs, let alone their evasions. It is very likely that the periodic silencing and evasion of MaLRs by ZNFs is not just a mechanism to control transposon propagation but also a fundamental driving force for the continuous evolution of MaLRs, particularly in primates.To firmly establish the continuous co-evolution model between MaLRs and ZNFs, we need four pieces of evidence. Firstly, we need to determine the evolutionary history of MaLRs, including the succession order and approximate activity period of each MaLR, which is the primary focus of current work. Secondly, each candidate ZNF should be present during the active expansion phase of its corresponding MaLR to control the potential genome instability caused by random insertions. Both ZNF430 and ZNF100 are retained in the descendant species of Simiiformes with conserved contact residues (Fig. S6), but they are not present in tarsier or bushbaby, which align with this expectation. The same criteria should apply to other anti-MaLR ZNFs as well.Thirdly, we need to determine the detailed full-length specificity profiles of these anti-MaLR ZNFs. While it is relatively easy to identify the specific binding sites of ZNF430 and ZNF100 within THE1B and THE1A, respectively, due to their shared contact residues (Zuo 2023), for other ZNFs containing long arrays of fingers, this is not the general case. In recent years, the application of high-throughput biophysical techniques such as Spec-seq on long ZNFs, including CTCF and ZFY, has demonstrated sufficient resolution and reproducibility to achieve this (Zuo et al. 2023).Lastly, the chronological reconstruction of driver mutations, which is commonly used in the study of cancer progression (Pon and Marra 2015; Maura et al. 2019), should also be useful in establishing the co-evolution between MaLRs and ZNFs. It is desirable to have molecular evidence about the intermediate elements between each MaLR and its successor, similar to Pre-THE1A and Int-THE1A, so that we can deduce the chronological order of mutation events between two adjacent MaLRs and demonstrate that the ZNF evasion event likely drove the new MaLR expansion. Note that the current model assumes MaLR accumulated mutations in a sequential manner without considering recombination between repeats, mosaic form, etc. (Carter et al. 2022), which still awaits further evidence.In addition to ZNFs, the internal gag gene might also be involved in the continuous evolution of MaLRs. The observation that each MaLR had a limited activity period in history suggests two things. Firstly, newly evolved MaLRs cannot facilitate the replication of ancestral MaLRs, even if the full-length sequences of those ancestral MaLRs still exist within the genome. It is probable that the Gag protein evolved to preferentially recognize and package its cognate viral RNA, similar to the mechanism known as cis preference in LINE-1 (Wei et al. 2001). Secondly, each MaLR eventually ceased replication in germline cells due to both silencing by ZNFs and accumulated defective mutations precluding transcription or packaging, which affected both autonomous replication and piggybacking on younger elements. It remains unclear whether the extinction of THE1A as the last MaLR member is related to this phenomenon. More experimental evidence is needed to confirm this hypothesis.

## Methods

### Visualization of MaLR distribution across representative species

The RepeatMasker data were directly downloaded from the UCSC genome browser, which included the following genomes: human (hg38.fa.out), gibbon (nomLeu3.fa.out), macaque (rheMac3.fa.out), marmoset (calJac4.fa.out), tarsier (tarSyr2.fa.out), mouse lemur (micMur2.fa.out), mouse (mm10.fa.out), pig (susScr3.fa.out), horse (equCab3.fa.out), elephant (loxAfr3.fa.out), armadillos (dasNov3.fa.out), and opossum (monDom5.fa.out). Only the MaLR family members were extracted and counted.

### Construction of phylogenetic tree

The LTR consensus sequence of each MaLR was downloaded from Dfam database (Model page in the website), and thus the internal indel information for each model was omitted. The tree was constructed using Geneious software with the following parameters: global alignment with free end gaps, transition/transversion ratio, Jukes–Cantor distance model, neighbor-joining method, and no outgroup. Figure S1 is the genetic distance table produced by the pairwise sequence alignment between different MaLR consensus sequences.

### Comparative genomic analysis

As shown in Fig. 3B, all MSTD, MSTC, MSTB2, MSTA1, and MSTB1 elements were extracted from RepeatMasker data (hg38.fa.out) using a minimum Smith-Waterman (S.W.) score of 100. The orthologous locations of these elements in tarsier and bushbaby were determined using the liftOver operations via the chain files hg38ToTarSyr2.over.chain and hg38ToOtoGar3.over.chain provided by UCSC genome browser, respectively. For each human MST element, its flanking regions were defined as 300bp sequences on each side of the element. If there was at least 200bp sequence present in the orthologous positions of the flanking regions on each side, we classified the human MST element as a properly aligned element. All elements were then grouped into entities based on shared repeatID. For each properly aligned entity, if its orthologous sequence in bushbaby or tarsier was longer than 50bp, it was considered to exist in bushbaby or tarsier. Based on the presence of MaLRs in bushbaby or tarsier, their relative insertion timings were determined accordingly.

### TinT inference

The TinT analysis is done using the interactive interface developed by Schmitz group (Churakov et al. 2010). RepeatMasker output (hg38.fa.out) was used as input, and the default parameters were applied.

### Identification of novel MaLR member in Tarsiiformes

To begin, RepeatModeler was utilized to identify all potential repeat families within the tarsier genome. One discovered family, known as family-40, contained the majority of MSTB1-related repeats; thus, this family of elements underwent further analysis using phylogenetic tree and comparative genomic analysis approaches. A group of elements within family-40 was found to be exclusively present in tarsiers but not in the human genome. The consensus sequence constructed from this group of elements has therefore been named MSTB1_TS.

Next, the RepeatMasker program and the consensus sequences of all MaLRs (including MSTB1_TS) were used to determine and quantify the presence of MaLRs in the mouse lemur, bushbaby, tarsier, and human genomes, respectively. The final counts of relevant MaLRs can be seen in Fig. 5A. At present, no new MaLR member has been discovered in the mouse lemur or bushbaby genome.

### Identification of intermediate elements between THE1B and THE1A

All full-length THE1A and THE1B elements (those elements that contain THE1-int sequences) annotated in RepeatMasker file (hg38.fa.align) were aligned against reference THE1A consensus sequence and inspected manually, especially at the loci related to structural variations in Fig. 6A. The annotated alignment file has been deposited in zenodo database as described in Data Availability section. Currently, only two elements (repeatID 4754186 and 2759773, see Fig. 6B) were found to lack the 204del feature within the putative ZNF100 binding sites. However, similar elements may exist in other simian genomes, potentially serving as precursors for Int-THE1A and other THE1A elements.

## Supplementary Material

vead072_Supp

## Data Availability

The supplemental data including estimation of the timing of insertion events through comparative genomics and THE1A sequences alignment with consensus are available through zenodo database with DOI: 10.5281/zenodo.10060190. The MSTB1_TS consensus sequence has been submitted to the Dfam database.
