# Supplementary material for: The successive emergence of ERVL-MaLRs in primates
Source: Virus Evol. 2023 Dec 4;9(2):vead072. doi: 10.1093/ve/vead072 (PMC10735291; doi:10.1093/ve/vead072)
Supplement: vead072_Supp [file vead072_supp.zip › suppl_data/Supplemental Information(1).pdf]

	THE1D	THE1C	THE1B	THE1A	MSTD	MSTC	MSTB2	MSTB1	MSTB	MSTA1	MSTA	MLTG1	MLT1N2	MLT1M	MLT1L	MLT1K	MLT1J2	MLT1J1	MLT1J	MLT1I
THE1D		0.069	0.116	0.168	0.420	0.416	0.334	0.281	0.208	0.296	0.089	0.704	0.821	0.866	0.968	0.726	0.656	0.728	0.761	0.675
THE1C	0.069		0.109	0.124	0.398	0.440	0.293	0.294	0.239	0.320	0.164	0.723	0.630	0.797	0.828	0.767	0.931	0.597	0.782	0.688
THE1B	0.116	0.109		0.087	0.407	0.427	0.334	0.331	0.255	0.309	0.164	0.845	0.888	0.708	0.876	0.658	0.769	0.589	0.762	0.601
THE1A	0.168	0.124	0.087		0.393	0.475	0.340	0.373	0.310	0.388	0.218	0.784	0.802	0.859	0.703	0.702	1.009	1.092	0.833	0.610
MSTD	0.420	0.398	0.407	0.393		0.193	0.238	0.268	0.301	0.183	0.297	0.692	0.746	0.921	0.613	0.622	0.612	0.655	0.561	0.751
MSTC	0.416	0.440	0.427	0.475	0.193		0.328	0.326	0.361	0.295	0.407	0.697	0.841	0.680	0.810	0.689	0.888	0.697	0.675	0.732
MSTB2	0.334	0.293	0.334	0.340	0.238	0.328		0.189	0.228	0.223	0.315	0.635	0.712	0.780	0.696	0.713	0.840	0.803	0.698	0.722
MSTB1	0.281	0.294	0.331	0.373	0.268	0.326	0.189		0.068	0.062	0.179	0.770	0.748	0.693	0.717	0.822	0.617	0.686	0.641	0.802
MSTB	0.208	0.239	0.255	0.310	0.301	0.361	0.228	0.068		0.108	0.118	0.655	0.715	0.770	0.704	0.692	0.741	0.669	0.641	0.756
MSTA1	0.296	0.320	0.309	0.388	0.183	0.295	0.223	0.062	0.108		0.230	0.798	0.699	0.861	0.673	0.668	0.612	0.612	0.678	0.743
MSTA	0.089	0.164	0.164	0.218	0.297	0.407	0.315	0.179	0.118	0.230		0.670	0.757	0.814	0.677	0.713	0.753	0.727	0.639	0.785
MLTG1	0.704	0.723	0.845	0.784	0.692	0.697	0.635	0.770	0.655	0.798	0.670		0.739	0.737	0.689	0.654	0.725	0.613	0.714	0.618
MLT1N2	0.821	0.630	0.888	0.802	0.746	0.841	0.712	0.748	0.715	0.699	0.757	0.739		0.639	0.886	0.639	0.602	0.613	0.601	0.784
MLT1M	0.866	0.797	0.708	0.859	0.921	0.680	0.780	0.693	0.770	0.861	0.814	0.737	0.639		0.675	0.522	0.837	0.759	0.741	1.017
MLT1L	0.968	0.828	0.876	0.703	0.613	0.810	0.696	0.717	0.704	0.673	0.677	0.689	0.886	0.675		0.262	0.474	0.461	0.252	0.679
MLT1K	0.726	0.767	0.658	0.702	0.622	0.689	0.713	0.822	0.692	0.668	0.713	0.654	0.639	0.522	0.262		0.584	0.579	0.429	0.813
MLT1J2	0.656	0.931	0.769	1.009	0.612	0.888	0.840	0.617	0.741	0.612	0.753	0.725	0.602	0.837	0.474	0.584		0.130	0.265	0.270
MLT1J1	0.728	0.597	0.589	1.092	0.655	0.697	0.803	0.686	0.669	0.612	0.727	0.613	0.613	0.759	0.461	0.579	0.130		0.260	0.282
MLT1J	0.761	0.782	0.762	0.833	0.561	0.675	0.698	0.641	0.641	0.678	0.639	0.714	0.601	0.741	0.252	0.429	0.265	0.260		0.305
MLT1I	0.675	0.688	0.601	0.610	0.751	0.732	0.722	0.802	0.756	0.743	0.785	0.618	0.784	1.017	0.679	0.813	0.270	0.282	0.305	
MLT1H2	0.773	0.709	0.732	0.859	0.763	0.682	0.696	0.719	0.729	0.677	0.618	0.295	0.680	0.825	0.632	0.490	0.474	0.523	0.500	0.617
MLT1H1	0.860	0.826	0.814	0.739	0.777	0.629	0.708	0.628	0.756	0.791	0.738	0.182	0.698	0.860	0.637	0.692	0.651	0.627	0.759	0.539
MLT1H	0.736	0.733	0.809	0.787	0.654	0.607	0.772	0.761	0.651	0.894	0.741	0.258	0.656	0.804	0.614	0.624	0.357	0.518	0.445	0.505
MLT1G3	0.704	0.701	0.636	0.790	0.756	0.825	0.715	0.740	0.658	0.752	0.767	0.062	0.692	0.683	0.662	0.735	0.689	0.601	0.600	0.596
MLT1G	0.774	0.851	0.850	0.734	0.687	0.740	0.787	0.732	0.773	0.741	0.883	0.155	0.823	0.802	0.696	0.704	0.651	0.599	0.731	0.552
MLT1F2	0.767	0.690	0.702	0.880	0.643	0.627	0.674	0.652	0.579	0.740	0.639	0.317	0.662	0.751	0.646	0.599	0.694	0.648	0.687	0.661
MLT1F1	0.829	0.659	0.857	0.778	0.638	0.653	0.743	0.651	0.634	0.733	0.695	0.247	0.641	0.729	0.686	0.756	0.597	0.591	0.715	0.630
MLT1F	0.914	0.788	0.782	0.856	0.718	0.681	0.707	0.806	0.660	0.787	0.642	0.211	0.764	0.671	0.664	0.631	0.621	0.577	0.597	0.593
MLT1E3	0.800	0.809	0.737	0.776	0.724	0.776	0.705	0.605	0.662	0.735	0.754	0.555	0.777	0.769	0.624	0.734	0.689	0.599	0.650	0.563
MLT1E2	0.735	0.845	0.937	0.800	0.668	0.588	0.647	0.771	0.631	0.716	0.717	0.496	0.701	0.684	0.741	0.777	0.675	0.680	0.682	0.616
MLT1E1A	0.843	0.800	0.737	0.804	0.668	0.862	0.698	0.689	0.685	0.732	0.711	0.457	0.820	0.783	0.676	0.648	0.671	0.655	0.771	0.608
MLT1E1	0.740	0.668	0.679	0.804	0.669	0.542	0.689	0.762	0.739	0.750	0.587	0.594	0.865	0.811	0.583	0.730	0.685	0.661	0.691	0.639
MLT1E	0.828	0.729	0.726	0.761	0.703	0.673	0.706	0.785	0.707	0.759	0.700	0.604	0.862	0.806	0.624	0.780	0.647	0.745	0.712	0.654
MLT1D	0.821	0.939	0.801	0.755	0.615	0.674	0.765	0.726	0.655	0.805	0.701	0.620	0.776	0.737	0.758	0.675	0.534	0.560	0.641	0.655
MLT1C2	0.630	0.678	0.683	0.694	0.485	0.591	0.624	0.670	0.638	0.647	0.644	0.659	0.652	0.775	0.612	0.900	0.579	0.714	0.505	0.565
MLT1C	0.557	0.631	0.649	0.676	0.428	0.452	0.601	0.599	0.522	0.546	0.649	0.702	0.632	0.821	0.636	0.627	0.575	0.632	0.737	0.637
MLT1B	0.640	0.621	0.559	0.590	0.408	0.434	0.461	0.593	0.594	0.460	0.580	0.640	1.022	0.791	0.610	0.639	0.675	0.710	0.653	0.629
MLT1A1	0.480	0.509	0.516	0.526	0.182	0.332	0.273	0.225	0.280	0.246	0.438	0.777	0.819	0.832	0.604	0.669	0.738	0.610	0.791	0.685
MLT1A0	0.505	0.525	0.603	0.578	0.234	0.262	0.262	0.252	0.319	0.269	0.366	0.630	0.696	0.888	0.554	0.706	0.590	0.620	0.693	0.569
MLT1A	0.605	0.532	0.545	0.546	0.383	0.296	0.322	0.299	0.361	0.311	0.365	0.640	0.709	0.705	0.561	0.626	0.578	0.628	0.595	0.573

	MLT1H2	MLT1H1	MLT1H	MLT1G3	MLT1G	MLT1F2	MLT1F1	MLT1F	MLT1E3	MLT1E2	MLT1E1A	MLT1E1	MLT1E	MLT1D	MLT1C2	MLT1C	MLT1B	MLT1A1	MLT1A0	MLT1A
THE1D	0.773	0.860	0.736	0.704	0.774	0.767	0.829	0.914	0.800	0.735	0.843	0.740	0.828	0.821	0.630	0.557	0.640	0.480	0.505	0.605
THE1C	0.709	0.826	0.733	0.701	0.851	0.690	0.659	0.788	0.809	0.845	0.800	0.668	0.729	0.939	0.678	0.631	0.621	0.509	0.525	0.532
THE1B	0.732	0.814	0.809	0.636	0.850	0.702	0.857	0.782	0.737	0.937	0.737	0.679	0.726	0.801	0.683	0.649	0.559	0.516	0.603	0.545
THE1A	0.859	0.739	0.787	0.790	0.734	0.880	0.778	0.856	0.776	0.800	0.804	0.804	0.761	0.755	0.694	0.676	0.590	0.526	0.578	0.546
MSTD	0.763	0.777	0.654	0.756	0.687	0.643	0.638	0.718	0.724	0.668	0.668	0.669	0.703	0.615	0.485	0.428	0.408	0.182	0.234	0.383
MSTC	0.682	0.629	0.607	0.825	0.740	0.627	0.653	0.681	0.776	0.588	0.862	0.542	0.673	0.674	0.591	0.452	0.434	0.332	0.262	0.296
MSTB2	0.696	0.708	0.772	0.715	0.787	0.674	0.743	0.707	0.705	0.647	0.698	0.689	0.706	0.765	0.624	0.601	0.461	0.273	0.262	0.322
MSTB1	0.719	0.628	0.761	0.740	0.732	0.652	0.651	0.806	0.605	0.771	0.689	0.762	0.785	0.726	0.670	0.599	0.593	0.225	0.252	0.299
MSTB	0.729	0.756	0.651	0.658	0.773	0.579	0.634	0.660	0.662	0.631	0.685	0.739	0.707	0.655	0.638	0.522	0.594	0.280	0.319	0.361
MSTA1	0.677	0.791	0.894	0.752	0.741	0.740	0.733	0.787	0.735	0.716	0.732	0.750	0.759	0.805	0.647	0.546	0.460	0.246	0.269	0.311
MSTA	0.618	0.738	0.741	0.767	0.883	0.639	0.695	0.642	0.754	0.717	0.711	0.587	0.700	0.701	0.644	0.649	0.580	0.438	0.366	0.365
MLTG1	0.295	0.182	0.258	0.062	0.155	0.317	0.247	0.211	0.555	0.496	0.457	0.594	0.604	0.620	0.659	0.702	0.640	0.777	0.630	0.640
MLT1N2	0.680	0.698	0.656	0.692	0.823	0.662	0.641	0.764	0.777	0.701	0.820	0.865	0.862	0.776	0.652	0.632	1.022	0.819	0.696	0.709
MLT1M	0.825	0.860	0.804	0.683	0.802	0.751	0.729	0.671	0.769	0.684	0.783	0.811	0.806	0.737	0.775	0.821	0.791	0.832	0.888	0.705
MLT1L	0.632	0.637	0.614	0.662	0.696	0.646	0.686	0.664	0.624	0.741	0.676	0.583	0.624	0.758	0.612	0.636	0.610	0.604	0.554	0.561
MLT1K	0.490	0.692	0.624	0.735	0.704	0.599	0.756	0.631	0.734	0.777	0.648	0.730	0.780	0.675	0.900	0.627	0.639	0.669	0.706	0.626
MLT1J2	0.474	0.651	0.357	0.689	0.651	0.694	0.597	0.621	0.689	0.675	0.671	0.685	0.647	0.534	0.579	0.575	0.675	0.738	0.590	0.578
MLT1J1	0.523	0.627	0.518	0.601	0.599	0.648	0.591	0.577	0.599	0.680	0.655	0.661	0.745	0.560	0.714	0.632	0.710	0.610	0.620	0.628
MLT1J	0.500	0.759	0.445	0.600	0.731	0.687	0.715	0.597	0.650	0.682	0.771	0.691	0.712	0.641	0.505	0.737	0.653	0.791	0.693	0.595
MLT1I	0.617	0.539	0.505	0.596	0.552	0.661	0.630	0.593	0.563	0.616	0.608	0.639	0.654	0.655	0.565	0.637	0.629	0.685	0.569	0.573
MLT1H2		0.230	0.259	0.332	0.296	0.317	0.349	0.338	0.606	0.583	0.590	0.555	0.514	0.591	0.566	0.636	0.522	0.640	0.598	0.579
MLT1H1	0.230		0.213	0.195	0.125	0.341	0.243	0.258	0.547	0.561	0.526	0.527	0.499	0.576	0.595	0.678	0.654	0.615	0.615	0.571
MLT1H	0.259	0.213		0.251	0.247	0.335	0.266	0.322	0.513	0.563	0.453	0.534	0.495	0.600	0.588	0.644	0.655	0.697	0.661	0.754
MLT1G3	0.332	0.195	0.251		0.055	0.390	0.277	0.315	0.610	0.591	0.529	0.586	0.549	0.585	0.535	0.608	0.770	0.623	0.619	0.688
MLT1G	0.296	0.125	0.247	0.055		0.360	0.274	0.327	0.615	0.568	0.496	0.499	0.509	0.618	0.770	0.630	0.658	0.720	0.669	0.703
MLT1F2	0.317	0.341	0.335	0.390	0.360		0.125	0.108	0.288	0.316	0.155	0.173	0.180	0.500	0.532	0.627	0.598	0.687	0.647	0.582
MLT1F1	0.349	0.243	0.266	0.277	0.274	0.125		0.083	0.378	0.379	0.353	0.329	0.331	0.583	0.535	0.600	0.569	0.688	0.614	0.596
MLT1F	0.338	0.258	0.322	0.315	0.327	0.108	0.083		0.371	0.399	0.269	0.256	0.325	0.548	0.537	0.667	0.642	0.588	0.614	0.631
MLT1E3	0.606	0.547	0.513	0.610	0.615	0.288	0.378	0.371		0.130	0.147	0.156	0.186	0.248	0.498	0.536	0.588	0.624	0.611	0.629
MLT1E2	0.583	0.561	0.563	0.591	0.568	0.316	0.379	0.399	0.130		0.213	0.222	0.220	0.125	0.254	0.283	0.599	0.505	0.617	0.575
MLT1E1A	0.590	0.526	0.453	0.529	0.496	0.155	0.353	0.269	0.147	0.213		0.036	0.040	0.296	0.479	0.565	0.543	0.592	0.608	0.644
MLT1E1	0.555	0.527	0.534	0.586	0.499	0.173	0.329	0.256	0.156	0.222	0.036		0.020	0.302	0.546	0.532	0.567	0.614	0.685	0.595
MLT1E	0.514	0.499	0.495	0.549	0.509	0.180	0.331	0.325	0.186	0.220	0.040	0.020		0.248	0.481	0.595	0.587	0.613	0.608	0.628
MLT1D	0.591	0.576	0.600	0.585	0.618	0.500	0.583	0.548	0.248	0.125	0.296	0.302	0.248		0.193	0.273	0.254	0.734	0.651	0.639
MLT1C2	0.566	0.595	0.588	0.535	0.770	0.532	0.535	0.537	0.498	0.254	0.479	0.546	0.481	0.193		0.075	0.140	0.391	0.286	0.266
MLT1C	0.636	0.678	0.644	0.608	0.630	0.627	0.600	0.667	0.536	0.283	0.565	0.532	0.595	0.273	0.075		0.083	0.333	0.280	0.204
MLT1B	0.522	0.654	0.655	0.770	0.658	0.598	0.569	0.642	0.588	0.599	0.543	0.567	0.587	0.254	0.140	0.083		0.304	0.217	0.170
MLT1A1	0.640	0.615	0.697	0.623	0.720	0.687	0.688	0.588	0.624	0.505	0.592	0.614	0.613	0.734	0.391	0.333	0.304		0.107	0.150
MLT1A0	0.598	0.615	0.661	0.619	0.669	0.647	0.614	0.614	0.611	0.617	0.608	0.685	0.608	0.651	0.286	0.280	0.217	0.107		0.059
MLT1A	0.579	0.571	0.754	0.688	0.703	0.582	0.596	0.631	0.629	0.575	0.644	0.595	0.628	0.639	0.266	0.204	0.170	0.150	0.059	

Figure S1 Distance map associated with phylogenetic tree in Figure 1

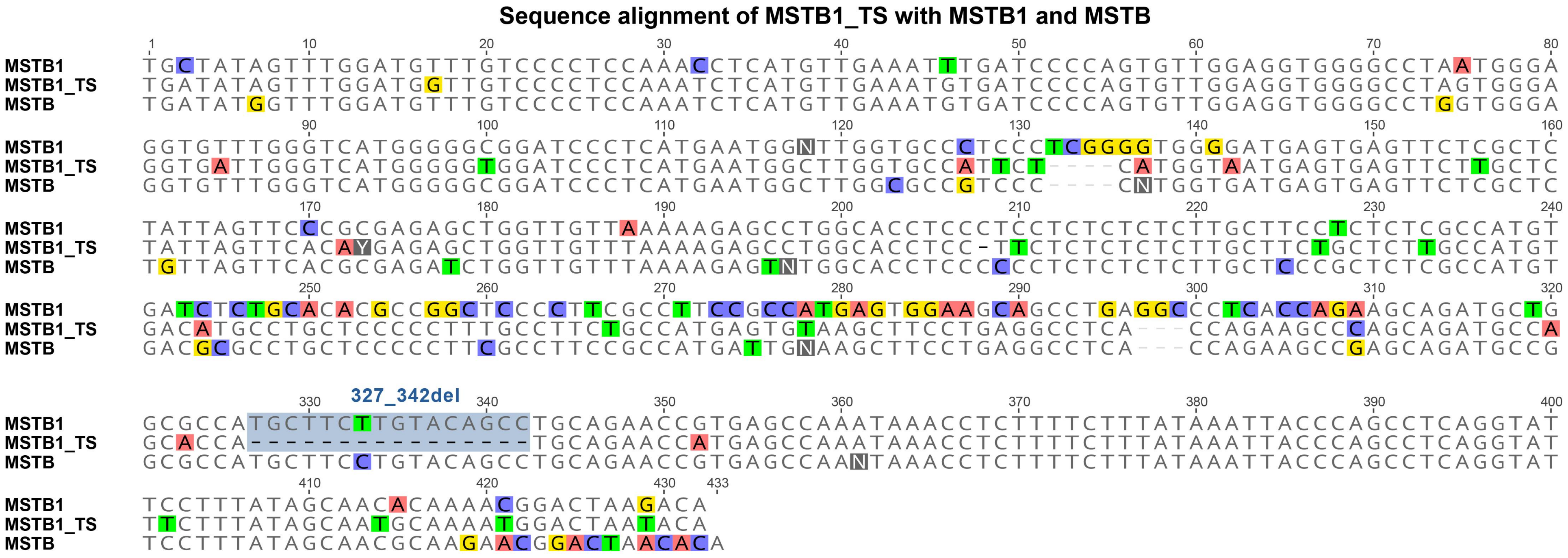

Figure S2 Full alignment of MSTB1_TS with MSTB1 and MSTB consensus sequences

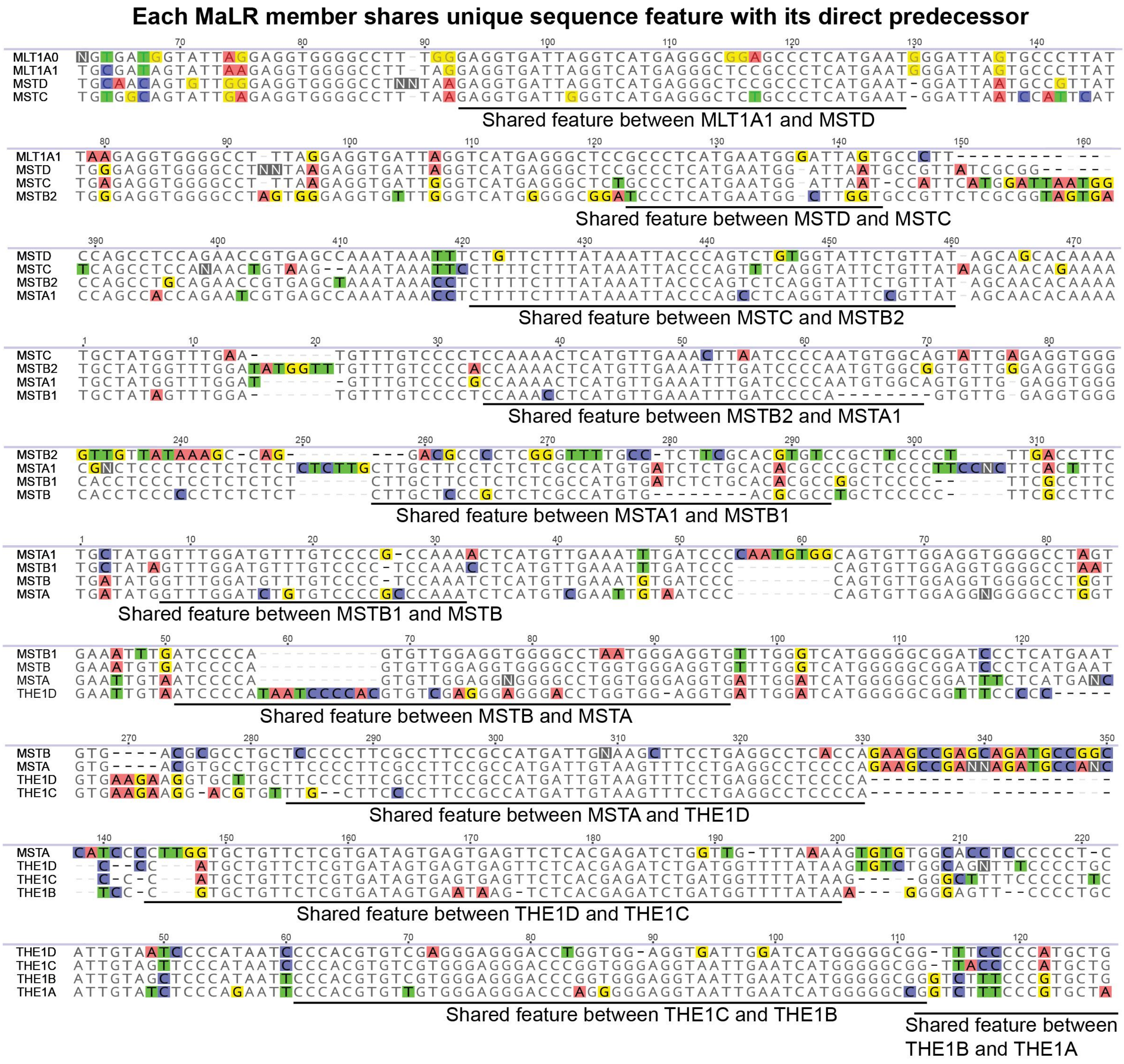

Figure S3 Uniquely shared sequence features between each MaLR and its direct predecessor

A

Alignment of left-hand arms of Precursor-THE1A elements in human genome and orthlogous species to THE1A consensus sequence

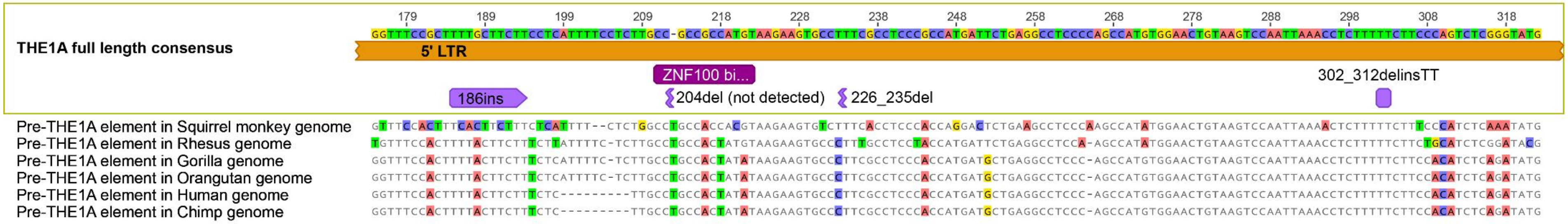

B

Alignment of right-hand arms of Precursor-THE1A elements in human genome and orthlogous species to THE1A consensus sequence

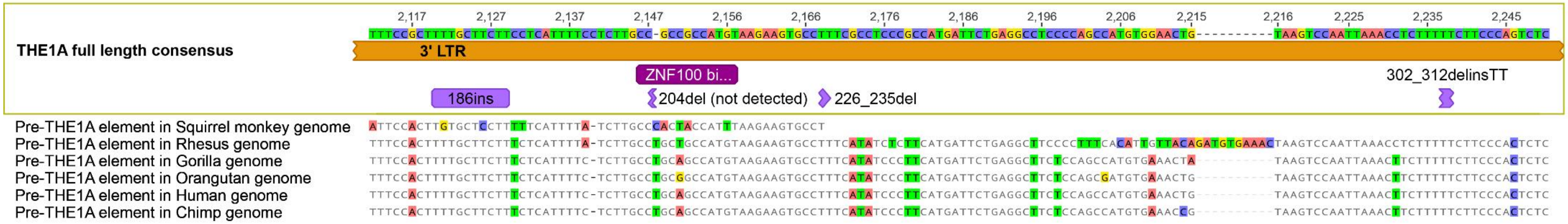

Figure S4 Alignment of left-hand and right-hand arms of Precursor-THE1A elements to THE1A consensus

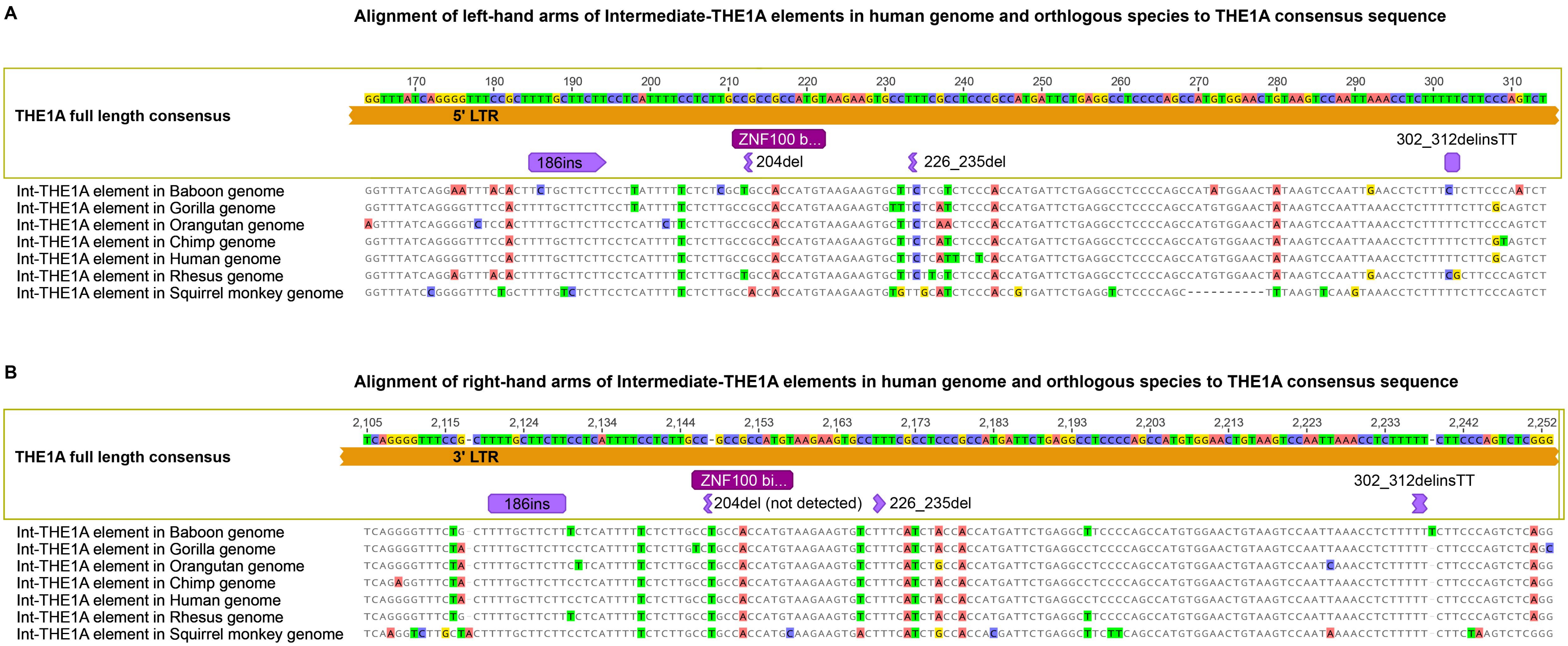

Figure S5 Alignment of left-hand and right-hand arms of Intermediate-THE1A elements to THE1A consensus

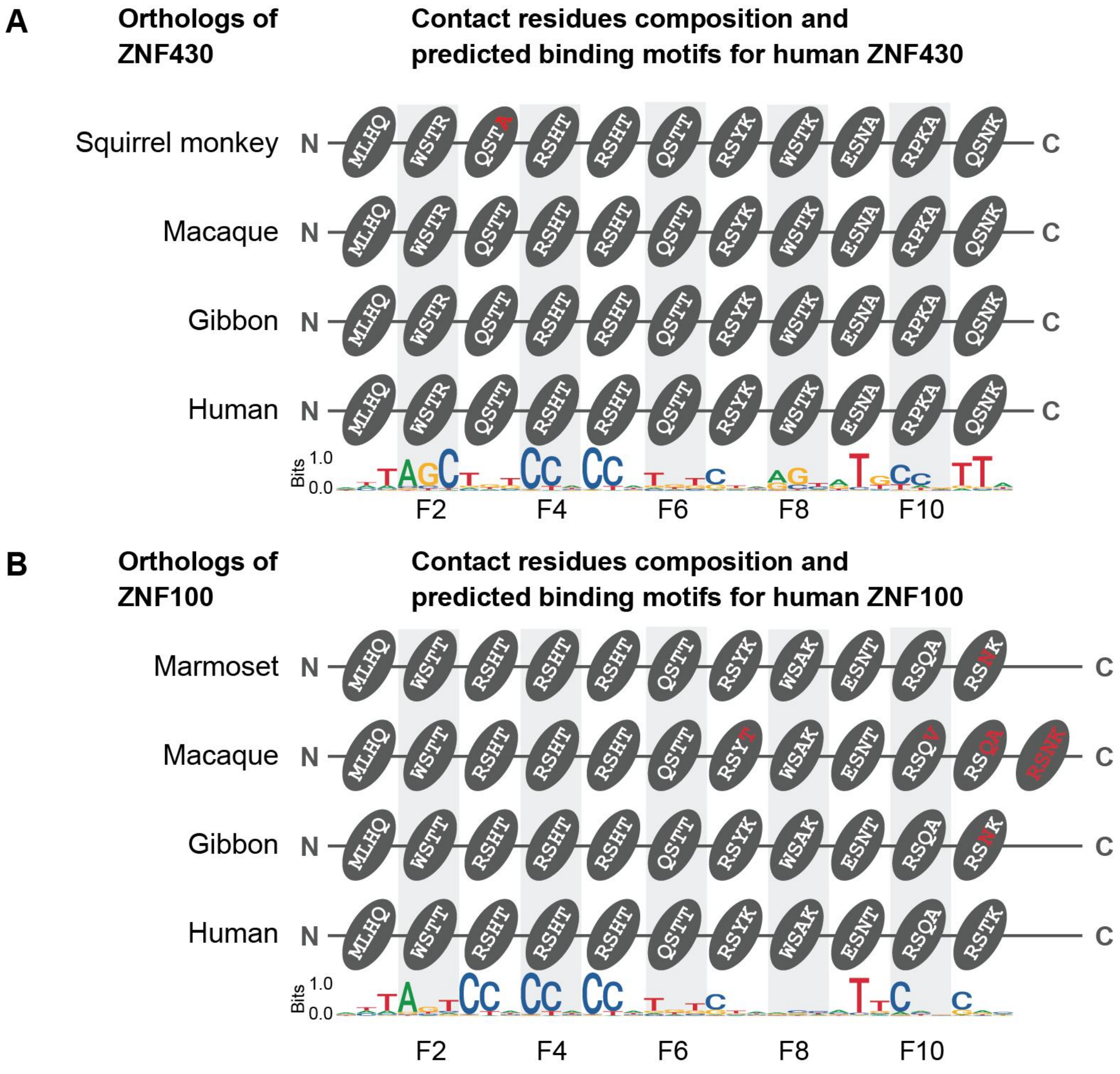

Figure S6 Contact residues composition for ZNF430/100 and their orthologs in Simiiformes
